# Design and Synthesis of Novel Squaraine-Based Fluorescent Probe for Far-Red Detection of Chymotrypsin Enzyme

**DOI:** 10.3390/molecules29061282

**Published:** 2024-03-14

**Authors:** Shekhar Gupta, Priyanka Balyan, Sai Kiran Mavileti, Shyam S. Pandey, Tamaki Kato

**Affiliations:** Graduate School of Life Science and Systems Engineering, Kyushu Institute of Technology, 2-4, Hibikino, Kitakyushu 808-0196, Japan; gupta.shekhar932@mail.kyutech.jp (S.G.); priyankabalyan1705@gmail.com (P.B.); m.c.saikiran.sai25@gmail.com (S.K.M.)

**Keywords:** homo-FRET, dye–peptide conjugate, chymotrypsin detection, far-red squaraine dye, fluorescent peptides, fluorescence microscopy

## Abstract

Chymotrypsin, a crucial enzyme in human digestion, catalyzes the breakdown of milk proteins, underscoring its significance in both health diagnostics and dairy quality assurance. Addressing the critical need for rapid, cost-effective detection methods, we introduce a groundbreaking approach utilizing far-red technology and HOMO-Förster resonance energy transfer (FRET). Our novel probe, SQ-122 PC, features a unique molecular design that includes a squaraine dye (SQ), a peptide linker, and SQ moieties synthesized through solid-phase peptide synthesis. Demonstrating a remarkable quenching efficiency of 93.75% in a tailored H_2_O:DMSO (7:3) solvent system, our probe exhibits absorption and emission properties within the far-red spectrum, with an unprecedented detection limit of 0.130 nM. Importantly, our method offers unparalleled selectivity towards chymotrypsin, ensuring robust and accurate enzyme detection. This pioneering work underscores the immense potential of far-red-based homo-FRET systems in enabling the sensitive and specific detection of chymotrypsin enzyme activity. By bridging the gap between cutting-edge technology and biomedical diagnostics, our findings herald a new era of enzyme sensing, promising transformative advancements in disease diagnosis and dairy quality control.

## 1. Introduction

Proteases are essential physiological enzymes that focus on creating straightforward and responsive assays applicable to disease diagnosis, therapy, and biological research. At specific sites along the polypeptide chain, proteases hydrolyze the amide bond, which plays a crucial role in the regulation of several physiological processes. These include cell proliferation, DNA replication, apoptosis, differentiation, immune responses, and hemostasis (coagulation) [1,2]. Chymotrypsin (CHT), classified as a serine protease, is commonly found in the digestive system of mammals [3]. Chymotrypsin cleaves the polypeptide substrate at the carboxy side of aromatic amino acids [4]. Chymotrypsin plays a significant role in regulating the digestion, necrosis, and apoptosis of dietary proteins [5,6,7]. Overdosed with chymotrypsin, the body releases histamine, which can cause allergic reactions [8]. In the fields of pathology and medicine, chymotrypsin exhibits anti-inflammatory effects and has proven effective in minimizing post-operative complications following cataract surgery [9]. Chymotrypsin is frequently employed as a representative protease due to its cost-effectiveness, easy accessibility, and recognition as a potential biomarker. To effectively detect chymotrypsin with high sensitivity, it is essential to develop a reliable fluorescent probe.

Although protease assay methods advanced rapidly, immunoassays utilizing antibodies for specific binding to target proteases were successfully developed and commercialized. These immunoassays are effective in quantitatively estimating protease levels [10]. However, they are not as suitable for mapping protease activity and correlating it with disease stages, as they primarily focus on quantifying protease quantity. To overcome this challenge, several techniques were widely used, including the use of suitable peptide substrates, optical detection (absorbance/fluorescence), and monitoring the increase or decrease in fluorescence utilizing Forster resonance energy transfer (FRET) [11,12]. Proteases and fluorogenic substrates interact to cause the peptide bond to be selectively cleaved, changing the fluorescence spectra of the enzymes and providing the basis for the detection of protease activity. Several effective FRET reporters based on small molecules have been developed so far [12,13,14]. While these probes showed high sensitivity in protease bioassays, the majority of them are based on a quencher (acceptor)-fluorophore (donor) system (hetro-FRET). Designing a donor–acceptor pair with ideal spectral characteristics poses a challenge when creating novel enzyme probes. Extensive utilization of fluorescent labels primarily functions in the visible or low wavelength [15,16,17] spectrum that can cause tissue damage; moreover, these fluorophores have blue-shifted emission bands that can interfere with cell autofluorescence. In this context, the incorporation of far-red and near-infrared (NIR) fluorophores not only boosts sensitivity by minimizing interference from biological autofluorescence but also facilitates effective bioimaging with enhanced depth penetration [18]. In optical imaging, fluorescent peptide-based probes are frequently employed, and multiple efforts are being made to explore the near-infrared imaging window of the spectrum [19]. Cyanine dyes emit at extended wavelengths, making them more suitable for use in tissues. However, their primary drawback lies in their rapid photobleaching rate [20,21]. In this context, NIR squaraine dye exhibits outstanding physical and chemical characteristics, including remarkably strong absorption bands, a high molar absorption coefficient, high quantum yield, good photostability, and favorable photoconductivity [22,23,24]. At the same time, the absorption and emission of the squaraine dye having a donor–acceptor–donor zwitterionic molecular framework can be tuned from the visible to the NIR wavelength region by judiciously selecting suitable donor moieties with varying π-conjugation [25]. In recent years, there has been a growing prevalence of utilizing the indole heterocycle as a donor unit in squaraine dyes. This trend has led to a diverse array of applications across different fields [26,27]. In a novel approach, Saikiran et al. designed a responsive sensor for human neutrophil elastase detection utilizing a squaraine dye-based aggregation-caused quenching mechanism, enabling real-time fluorescence monitoring of enzyme activity [28]. This design allows for real-time monitoring of enzyme activity through fluorescence changes, offering a rapid and cost-effective method for detection.

Top of Form

**Scheme 1:**
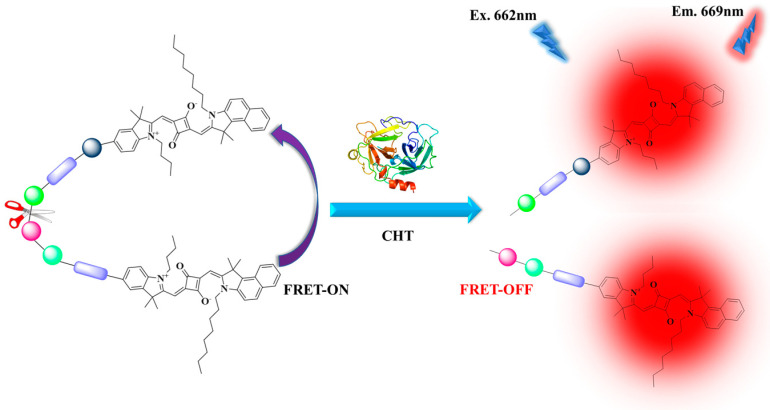
Schematic representation of chymotrypsin detection by fluorescence turns off/on mechanism due to homo-FRET.

To detect chymotrypsin in the far-red region, we designed and synthesized a novel dye–peptide conjugated probe (SQ-122 PC) based on the homo-Förster resonance energy transfer (homo-FRET) process. The design of the probe involves the incorporation of two far-red-sensitive squaraine dye molecules with a suitable peptide sequence present at each terminal to enable homo-FRET, and upon cleavage by chymotrypsin, the homo-FRET is off, leading to a pronounced enhancement of the fluorescence signal in the far-red region (Scheme 1). We achieved a highly sensitive detection platform capable of detecting chymotrypsin activity at extremely low concentrations. Furthermore, the probe demonstrates exceptional selectivity, exhibiting minimal interference from other proteases commonly encountered in complex biological samples.

## 2. Results and Discussion

### 2.1. Design and Synthetic Route

**Figure 1:**
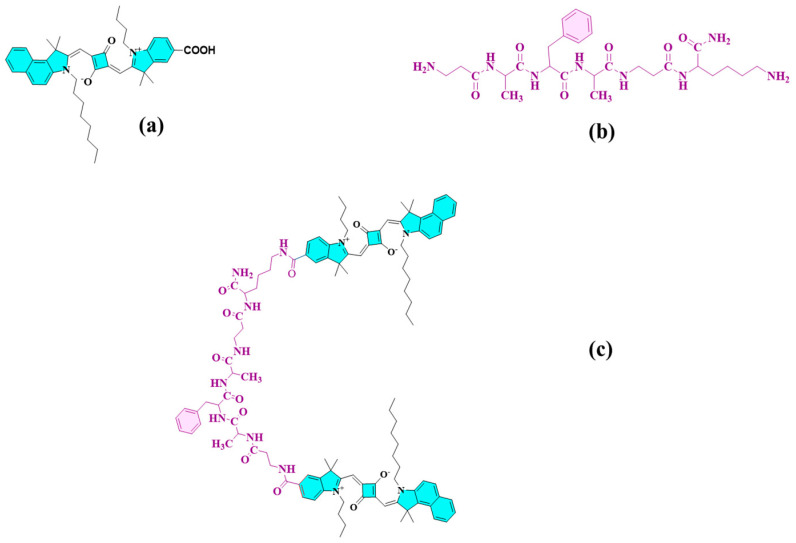
Chemical structure of unsymmetrical squaraine dye (SQ-122) (**a**), chymotrypsin enzyme specific peptide (**b**) dye–peptide conjugate probe (SQ-122 PC) (**c**).

**Scheme 2:**
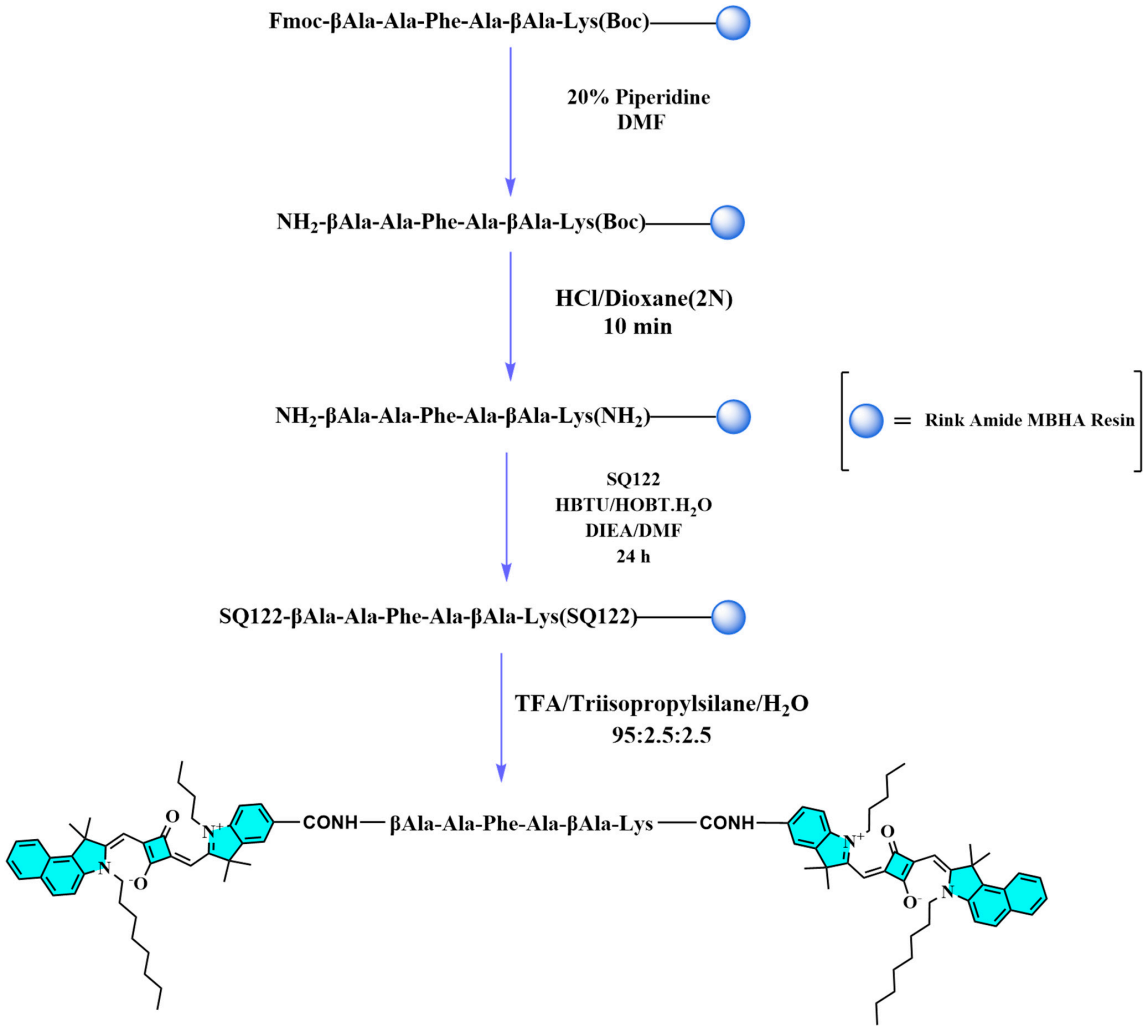
Scheme for the synthesis of dye peptide conjugate SQ122-PC.

The newly proposed peptide–dye conjugate distinctly shows the attachment of two squaraine dye molecules at both ends of the peptide terminals. As a result of its far-red fluorescence emission and its favorable interactions with frequently utilized model proteins, including human serum albumin and bovine serum albumin, squaraine dye was selected as the model dye [29,30,31,32]. The squaraine dye’s chain length plays a pivotal role in its behavior in aqueous environments, influencing aggregate formation. Specifically, shorter chain lengths are prone to forming both H-aggregates and J-aggregates, whereas longer alkyl chain substitutions, such as octyl and dodecyl, are observed to mitigate this aggregation through steric hindrance [33]. The lipophilicity of squaraine dye is increased by longer alkyl chains, which also have a hydrophobic effect that slightly lowers the chance of aggregate formation [34]. So, this led to the design and synthesis of benzo[e]indole squaraine dye (SQ-122), as shown in Figure 1a, with an octyl chain substituted at the N-terminal of the benzo-indole ring. The fluorescent probe SQ-122 PC was synthesized by solid-phase peptide synthesis, as shown in Scheme 2.

It was reported that chymotrypsin, a member of the superfamily serine proteases, cleaves peptide bonds that are on the C-terminal side of an amide linkage, which contains an aromatic side chain such as tyrosine (Tyr), phenylalanine (Phe), and tryptophan (Trp). Taking this point into consideration, we designed Ala-Phe-Ala as the target tripeptide along with β-Alanine (β-Ala) as a spacer to facilitate the accessibility of the chymotrypsin enzyme to the designed probe. On the other hand, lysine (Lys) was introduced for the side chain binding of the terminal squaraine dye. The construction of the probe SQ-122 PC is based on solid-phase peptide synthesis. The initial step was the introduction of Fmoc-Lys (Boc)-OH to Rink Amide MBHA Resin, resulting in Fmoc-Lys (Boc)-Resin. Following this, solid-phase peptide synthesis was utilized to gradually synthesize a linear peptide strain.

The incorporation of β-Ala and Lys groups serves as a spacer and provides a unique advantage in a FRET probe design. Moreover, the free amine of Lys acts as a binding group to the free COOH group of the dye (SQ-122). The synthesis process commenced with the resin-supported peptide (Fmoc-βAla-Ala-Phe-βAla-Lys(Boc)-Resin) undergoing the deprotection of the Fmoc group via treatment with 20% piperidine, followed by exposure to HCl/Dioxane (2 M) for 10 min to remove the Boc group. Following deprotection, the elongated peptide was then conjugated with SQ-122 at a molar ratio of 2.2:1 using HOBt/HBTU as a coupling reagent. Following this, the resin-supported peptide was detached from the resin using a cleavage cocktail containing TFA, triisopropylsilane, and H_2_O in a ratio of 95:2.5:2.5. The dye–peptide conjugate obtained after cleavage was precipitated with ether in an ice bath and further purified using silica gel column chromatography with a solvent system consisting of chloroform and methanol (9:1). This purification process yielded the dye–peptide conjugate in a satisfactory yield. The resulting compound exhibited a distinctive dark reddish color, indicating the successful formation of the conjugate. The verification of the synthesis success was achieved through Time of Flight (TOF) mass spectrometry analysis, which revealed measured values of 1858.10 [M + H^+^] and 1880.07 [M + Na^+^] (calculated 1857.07137). This analytical confirmation confirms the precise synthesis of the dye–peptide conjugate, demonstrating the efficacy of the synthetic methodology employed in the conjugation process. Further details on the synthesis process, reaction conditions, and analytical results, including NMR and mass spectrometry data, are comprehensively documented in the Supporting Information (Section S1, Scheme S1).

### 2.2. Photophysical Properties of Dye and Dye–Peptide Conjugate

SQ-122 normalized electronic absorption spectra in dimethyl sulfoxide (DMSO) solution exhibits a vibrionic shoulder at 620 nm and a prominent electronic absorption peak with an absorption maximum (λ_abs_) at 667 nm. In contrast, the emission spectrum of SQ-122 features a fluorescence emission peak at 682 nm, resulting in a relatively modest Stokes shift of 15 nm. This limited Stokes shift is characteristic of SQ-type molecules, indicating that the structural configurations of the dye’s ground and excited states are remarkably similar [35]. Table 1 displays the values of the molar extinction coefficients (ε), Stoke shifts (Δ), absorption maxima (λ_abs_) and emission maxima (λ_em_), and molar extinction coefficients (ε) of SQ-122 standalone dye and SQ-122 PC at a 5 μM solution in DMSO and H_2_O (30% DMSO). These data elucidate the optical properties of SQ-122, underscoring the dye’s stable chemical structure across different states and highlighting its potential for applications in fluorescence-based detection and imaging techniques.

**Figure 2:**
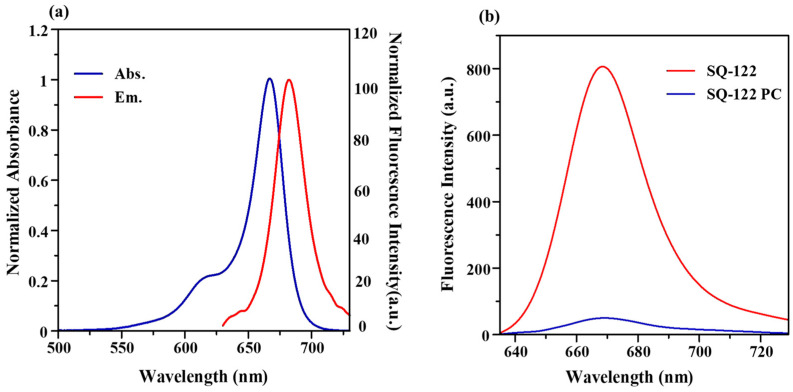
(**a**) Normalized absorbance and normalized fluorescence intensity spectra of SQ-122 in DMSO (5 μM). (**b**) Fluorescence emission spectra of SQ-122 (5 μM) and the probe, SQ-122 PC (5 μM), both in H_2_O (30% DMSO as co-solvent). The instrumental setting of slit width for the excitation and emission was both set at 10 nm, respectively.

For a FRET to occur, the excitation spectrum of the acceptor and the emission spectrum of the donor should overlap. In the case of homo-FRET, the energy transfer occurs between two identical fluorophores, provided they have an overlap between the excitation and emission spectrum [36]. In Figure 2a, it is evident that the excitation (absorption spectrum) and emission spectrum of the SQ-122 fluorophore exhibit a spectral overlap owing to the very small Stokes shift. Therefore, SQ-122 can be considered a suitable fluorophore for the design of the homo-FRET probe. Furthermore, homo-FRET involves energy transfer among identical fluorophores but does not impact the spectral characterization of the fluorophore and the probe [37]. It can be seen from the absorption spectra shown in Figure S1a that the absorption peak of SQ-122 and the probe SQ-122 PC appearing at the absorption maximum (λ_max_) in DMSO are the same (669 nm), suggesting that the incorporation of peptide does not alter the basic spectral behavior of the dye molecule. However, in an H_2_O (30% DMSO) medium, the absorption spectra of SQ-122 PC (Figure S1b) exhibit a noticeable shoulder peak at 616 nm and a blue-shifted absorption peak at 662 nm, suggestive of dye aggregation. Such aggregation tendencies in squaraine dyes, leading to either blue-shifted H-aggregates or red-shifted J-aggregates, are influenced by their planar molecular structure and environmental factors [38]. The quantum yield for the dye was determined to be 0.16%.

Interestingly, at a similar concentration (5 μM) and in the same solvent (H_2_O, 30% DMSO), the fluorescence intensity of SQ-122 PC is significantly lower than that of the pure dye SQ-122. The observation indicates that fluorescence quenching takes place when SQ-122 is linked with the examined peptide (SQ 122-PC), as shown in Figure 2b. The presence of this quenching effect suggests the occurrence of Förster resonance energy transfer (FRET) within the homo-dye-labeled probe, attributed to the minimal Stokes shift characteristic of the system. In such instances, FRET-mediated quenching is commonly observed, facilitated by a substantial overlap between the absorption and emission spectra of the dye, aligning with the empirical observations made in this study. Based on the peak fluorescence intensities of the dye alone (SQ-122) and the dye–peptide conjugate (SQ-122 PC), it was determined that the fluorescence quenching efficiency in H_2_O (30% DMSO) reached a calculated value of 93.75%.

### 2.3. Enzymatic Hydrolysis of SQ-122 PC with Chymotrypsin

**Figure 3:**
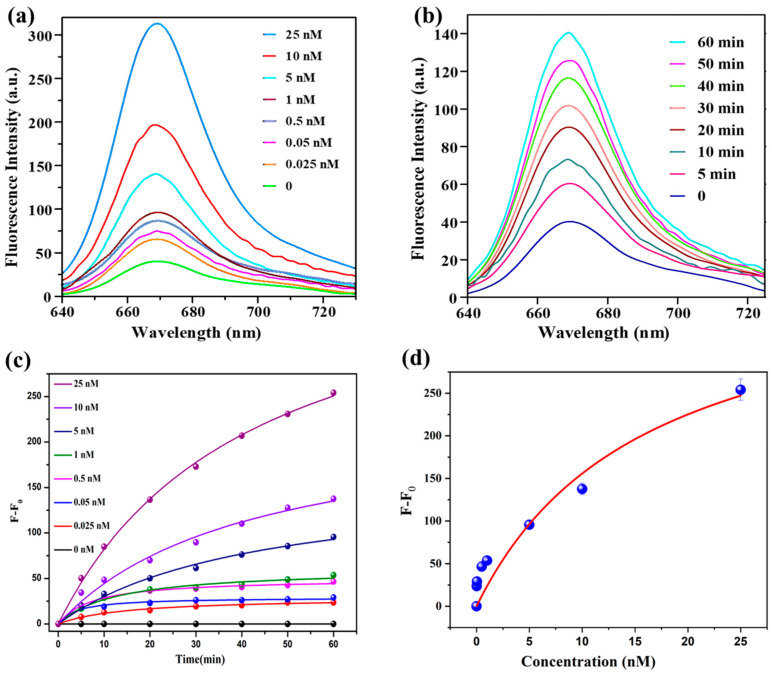
(**a**) Concentration-dependent fluorescence spectra of SQ-122 PC (5 μM) in H_2_O (30% DMSO) at 37 °C for 60 min with the addition of 0 to 25 nM of chymotrypsin. (**b**) Time-dependent fluorescence spectra of SQ-122 PC (5 μM) in H_2_O (30% DMSO) at 37 °C for 60 min with the addition of 5 nM of chymotrypsin. (**c**) Plot of difference in fluorescence intensity (F − F_0_) vs. time. (**d**) Plot of difference in fluorescence intensity (F − F_0_) vs. concentration of enzyme. (The instrumental setting of slit width for the excitation and emission was both set at 10 nm, respectively).

The SQ-122 PC probe displays a weak fluorescence due to the homo-FRET quenching effect, as indicated in Figure 2b. However, following incubation with chymotrypsin, a notable increase in fluorescence at 669 nm is observed. Different concentrations of chymotrypsin in PBS (pH 7.4, 0.1 mM) (0–25 nM) were added to 5 μM of SQ-122 PC in H_2_O (30% DMSO) and incubated for 60 min. There was a pronounced recovery of quenched fluorescence of the dye after the addition of the chymotrypsin enzyme, as shown in Figure 3a. This pronounced enhancement of the fluorescent signal can be attributed to the enzymatic hydrolysis of the SQ-122 PC that leads to the physical separation of FRET pairs, subsequently increasing the fluorescence intensity. Upon enzyme action, the peptide is cleaved, disrupting the energy transfer process between two squaraine dyes, resulting in a measurable change in fluorescence intensity. An increase in fluorescence intensity can be observed at the emission peak of the fluorophore, correlating directly with the enzyme’s activity level. Around an eightfold enhancement in the fluorescence signal upon the addition of 25 nM of chymotrypsin as compared to the absence of the enzyme and 5 nM of chymotrypsin led to a threefold enhancement Figure 3b. Moreover, the addition of 0.5 nM of chymotrypsin leads to a twofold enhancement in the fluorescent signal within 60 min, as shown in Figure S2. In Figure 3c,d F_0_ represents the initial fluorescence intensity in the absence of the enzyme, while F denotes the fluorescence intensity at a specific time point after the addition of the enzyme.

**Figure 4:**
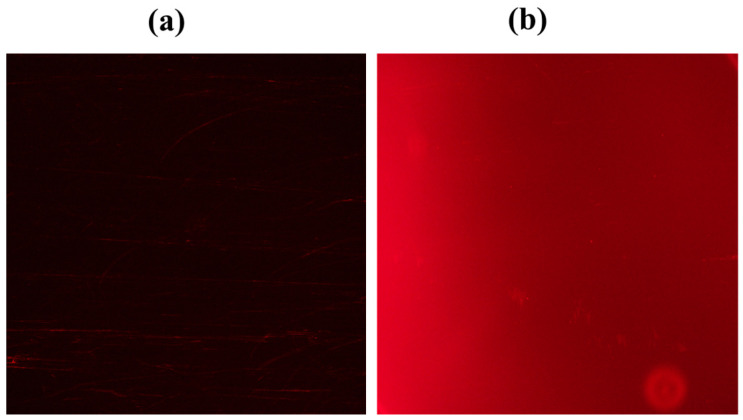
Fluorescence microscopy images of (**a**) SQ-122 PC (5 μM) in H_2_O (30% DMSO) and (**b**) SQ-122 PC (5 μM) in H_2_O (30% DMSO) pre-incubated with 25 nM of chymotrypsin at 37 °C for 60 min.

In our study, we explored the fluorescence properties of SQ-122 PC under different conditions using fluorescence microscopy [39,40], focusing on the impact of chymotrypsin. We compared fluorescence images obtained for SQ-122 PC under two conditions (Figure 4a,b). In condition (a), the fluorescence intensity of SQ-122 PC in the absence of an enzyme is negligible. However, after pre-incubation with chymotrypsin (condition b), we noted a significant increase in fluorescence emission. This enhancement in the fluorescence signal suggests the occurrence of FRET off, indicating a change in the energy transfer dynamics within the dye–peptide system.

### 2.4. Validation of the Peptide Cleavage by Chymotrypsin

**Figure 5:**
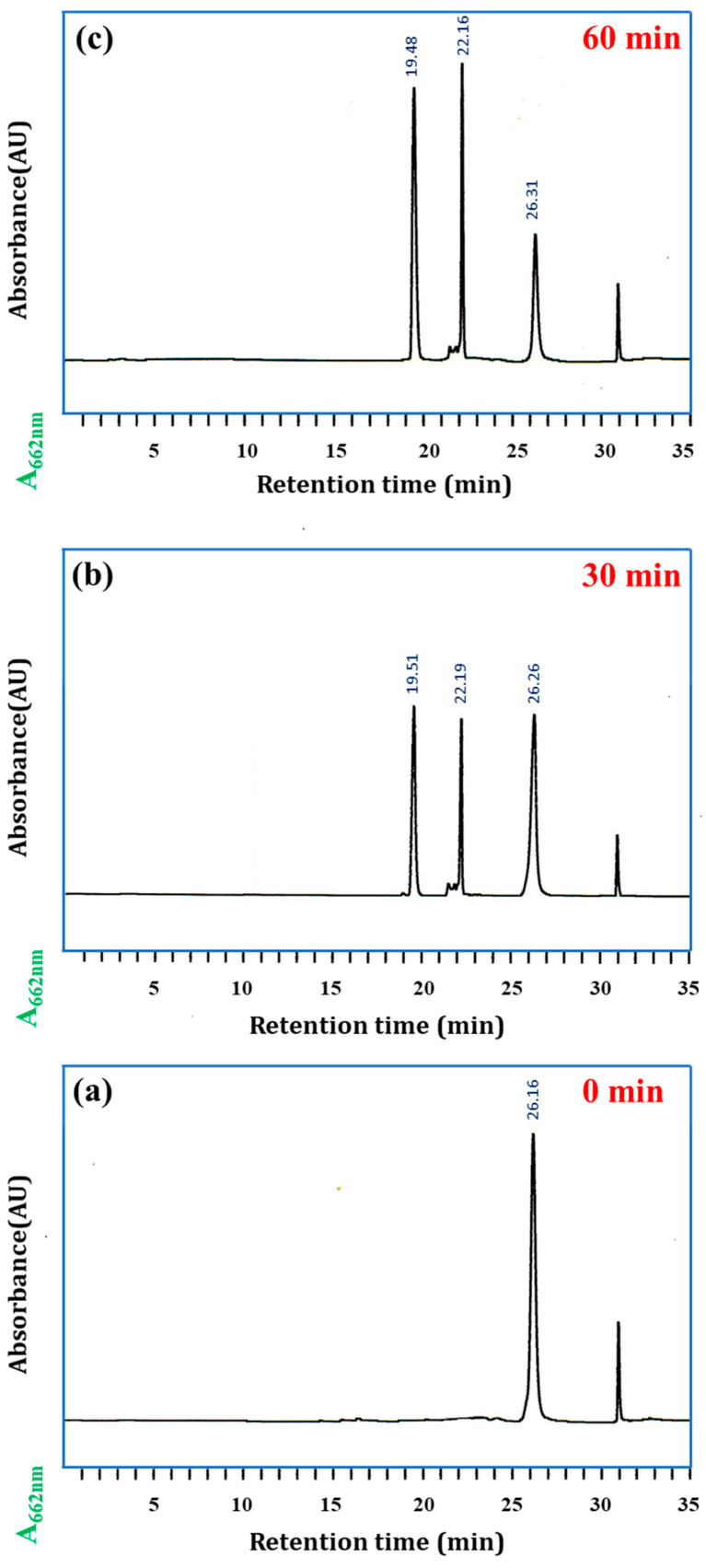
HPLC chromatogram that confirms the enzymatic cleavage of SQ-122 PC with chymotrypsin. (**a**) SQ-122 PC (10 μM) in H_2_O with 30% DMSO (0 min). (**b**) SQ-122 PC and chymotrypsin (10 nM) in H_2_O (30% DMSO) incubated at 37 °C for 30 min. (**c**) SQ-122 PC and chymotrypsin (10 nM) in H_2_O (30% DMSO) incubated at 37 °C for 60 min. The mobile phase was acetonitrile/water, and the flow rate was 1.0 mL/min.

To elucidate the enzymatic hydrolysis effects of chymotrypsin (CHT) on the SQ-122 PC probe and its subsequent impact on emission spectral characteristics, a comprehensive study employing high-performance liquid chromatography (HPLC) and high-resolution mass spectrometry (HRMS) was conducted. Figure 5 depicts the HPLC peaks observed for SQ-122 PC alone and after pre-incubation with CHT in H_2_O (30% DMSO) after 30 min and 60 min. The molecular ion peak at *m*/*z* 1858.08 corresponds to [M + H]^+^ of SQ-122 PC, and a single peak confirms the very high purity of the synthesized probe. In contrast, the HRMS spectrum obtained from the reaction mixture of probe SQ-122 PC with CHT reveals a prominent peak at *m*/*z* = 948.52 [M + H]^+^ and 929.54 [M + H]^+^ (Figure S3), suggesting the hydrolysis of the substrate that led to the formation of two unsymmetrical fragments generated by the cleavage of the Phe-Ala peptide bond. In the HPLC chromatogram, the SQ-122 PC and the resulting reaction products were detected at retention times of 26.26 min, 22.19 min, and 19.51 min, respectively. Notably, the emergence of new peaks at 22.19 min/22.16 min and 19.51 min/19.48 min (illustrated in Figure 5b,c) corresponds to the formation of the two unsymmetrical fragments, SQ122—βAla-Ala-Phe and Ala- βAla-Lys-SQ122, post-enzymatic action by chymotrypsin. This detailed analysis via HPLC and HRMS not only confirms the efficacy of the probe in substrate detection but also provides crucial insights into the precise cleavage sites and resultant fragments generated upon interaction with the target enzyme. Such investigations not only enhance our understanding of enzymatic processes but also lay a foundation for the development of advanced enzyme detection techniques and tailored molecular probes for diverse analytical and diagnostic applications [41].

### 2.5. Enzyme Selectivity of the Probe SQ-122 PC

**Figure 6:**
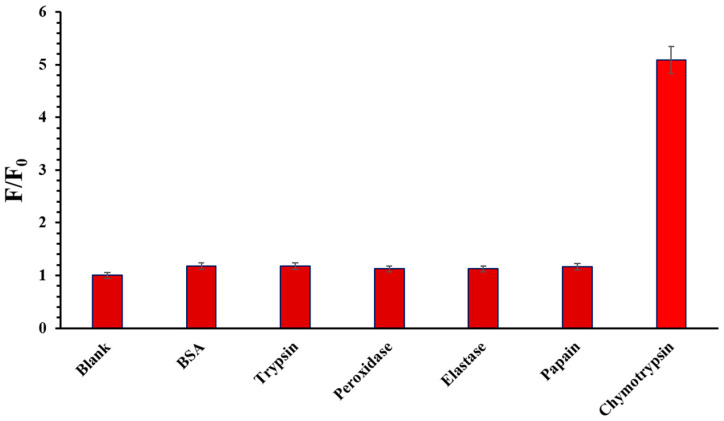
Fluorescence response (F/F_0_) of the probe towards different potential species (chymotrypsin, papain, pancreatic elastase, peroxidase from horseradish, trypsin, and BSA) of concentration 25 nM.

The selectivity studies of chymotrypsin in the presence of different enzymes involve investigating the enzyme’s ability to selectively recognize and cleave specific substrates in the presence of other enzymes. Chymotrypsin, papain, elastase pancreatic, peroxidase from horseradish, trypsin, and BSA (concentration of every enzyme at 25 nM) were added to a 5 μM solution of the probe in H_2_O (30% DMSO) and incubated at 37 °C for 60 min. Figure 6 illustrates that the emission intensity at 669 nm exhibited minimal variation in the presence of potential competitive enzymes. However, a significant increase in emission intensity was observed following the introduction of chymotrypsin. However, a significant increase in emission intensity was observed following the introduction of chymotrypsin. This pronounced change underlines the probe’s exceptional specificity towards chymotrypsin, showcasing its capability to selectively interact with and respond to the presence of this particular enzyme amidst a background of competing enzymatic species.

### 2.6. Sensitivity of the Probe, SQ-122 PC, to Chymotrypsin

**Figure 7:**
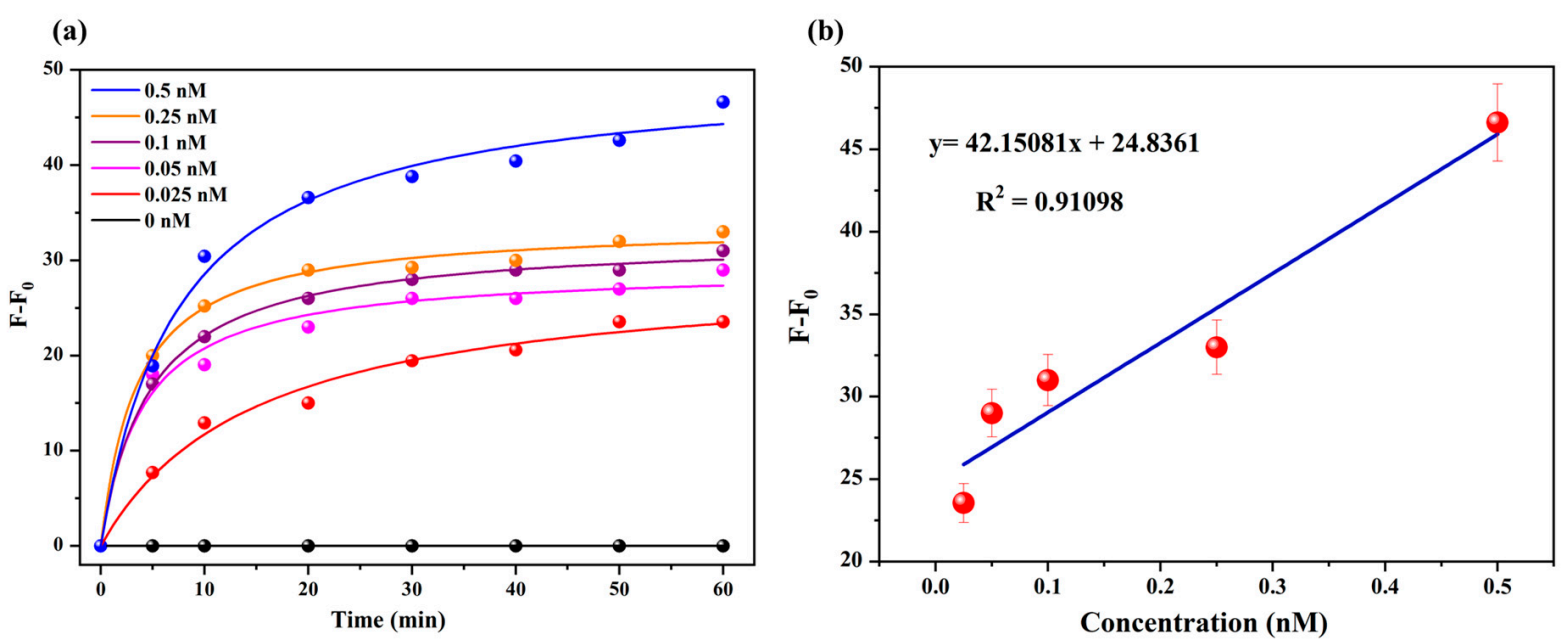
(**a**) The difference in fluorescence intensity with time with the addition of 0, 0.025, 0.05, 0.1, 0.25, and 0.5 nM of chymotrypsin in 5 μM of probe in H_2_O (30% DMSO) incubated at 37 °C for 60 min. (**b**) Linear correction curve of difference in fluorescence intensity at 669 nm against chymotrypsin concentration.

To examine the sensitivity of SQ-122 PC, different concentrations of chymotrypsin (0, 0.025 nM, 0.05 nM, 0.1 nM, 0.25 nM, and 0.5 nM) were introduced to SQ-122 PC, solutions (5 μM) in H_2_O (30% DMSO) at 37 °C for 60 min. Subsequently, the fluorescent response at 669 nm was recorded. Figure 7b illustrates a linear plot representing each sample’s difference in fluorescence intensities alongside their variable concentrations of the enzymes. The linear regression equation was y = 42.15081x + 24.8361, R^2^ = 0.91098. This analysis revealed a remarkable limit of detection (LOD) for chymotrypsin at 130 pM, highlighting the assay’s exceptional sensitivity. In comparison to probes employed in numerous prior investigations, SQ-122 PC emerges as a highly efficient and effective probe for chymotrypsin detection. A detailed comparison presented in Table 2 showcases the performance of SQ-122 PC relative to other methodologies reported in the literature for chymotrypsin detection. Our findings demonstrate comparable or even superior limits of detection (LOD) when juxtaposed with existing approaches, with the added advantage of enabling detection in the far-red spectral region, distinguishing our method from conventional techniques.

## 3. Materials and Methods

### 3.1. Reagents and Instruments

All the chemicals, solvents, and reagents for synthesis and photophysical characterization are of analytical or spectroscopic grade and used as received. All Fmoc-protected amino acids, Rink amide MBHA resin, piperidine, *O*-(1H-benzotriazol-1-yl)-*N*,*N*,*N*′,*N*′-tetramethyluronium hexafluorophosphate (HBTU), 1-hydrolxy-1Hbenzotriazole hydrate (HOBt·H_2_O), *N*,*N*-diisopropylethylamine (DIEA), 2,2,2-trifluoroacetic acid (TFA), and 4 M HCl/Dioxane were purchased from Watanabe Chemical Industries, Ltd. (Hiroshima, Japan) α-chymotrypsin from bovine pancreas, α-trypsin from bovine pancreas, elastase pancreatic, peroxidase from horseradish, bovine serum albumin, and papain were obtained from Sigma-Aldrich Co., LLC, Tokyo, Japan. All solvents and other reagents were ordered from Wako Pure Chemical Industries, Ltd., Osaka, Japan. Deionized water was acquired using a Milli-Q Plus system manufactured by Millipore. Synthesized unsymmetrical squaraine dye and dye intermediates were analyzed by TOF/FAB–mass spectroscopy in positive ion monitoring mode and nuclear magnetic resonance spectroscopy (NMR 500 MHz for 1 H NMR) for structural elucidation. Utilizing a UV–visible–NIR spectrophotometer (JASCO, Tokyo, Japan, V-530 UV/VIS spectrophotometer), the electronic absorption spectra in the solution were determined. A fluorescence emission spectrometer (JASCO FP-6600 spectrophotometer) was employed to record the fluorescence emission spectra. Analytical high-performance liquid chromatography (HPLC) was performed with an Xterra MS C8-5 μm column (4.6 × 150 mm; made by Waters) installed on Hitachi L-7100 (Tokyo, Japan) equipment. A linear gradient of solvent B in solvent A, ranging from 0% to 50% over 15 min, was used in the mobile phases, which were 0.1% trifluoroacetic acid (TFA) in H_2_O (designated as solvent A) and 0.1% TFA in acetonitrile (designated as solvent B). The flow rate was 1.0 mL/min. For detection, absorbance at 220 nm and 662 nm was used. The EYELA SLI-400 has been used for the incubation process of the samples. Fluorescence microscopy (ECLIPSE Ts2-FL, Nikon, Tokyo, Japan) captured fluorescence images of the probe SQ-122 PC with and without enzymes. The excitation wavelength was 580 nm.

### 3.2. Spectroscopic Measurements

A stock solution of SQ-122 PC (100 μM) in DMSO was made and used upon dilution with DMSO and H_2_O (pH 7.2, 30% DMSO). The stock solution of α-chymotrypsin bovine pancreas was 100 μM in PBS (pH 7.2, 0.1 M) and was used upon dilution. Similarly, for other enzymes, the concentration of the stock solution was 100 μM in PBS (pH 7.2, 0.1 M). The fluorescence reaction of SQ-122 PC (5 μM) to CHT was evaluated in H_2_O (pH 7.2, 30% DMSO), as outlined below. The reaction mixture with a total volume of 3 mL containing the probe and the enzyme was incubated at 37 °C, and the fluorescence response at 669 nm (both excitation and emission slit widths were set to 10 nm) was taken after 5 min and, after that, every 10 min for 60 min. The concentration of chymotrypsin in the cuvette was 0.025 nM, 0.05 nM, 0.5 nM, 1 nM, 5 nM, 10 nM, and 25 nM. The volume of the reaction mixture was adjusted such that the final concentration of the probe was 5 μM. To facilitate a comparative analysis of fluorescence intensity changes, a baseline intensity, denoted as F_0_, was established by measuring the fluorescence of SQ-122 PC at 5 μM in H_2_O (30% DMSO) at 669 nm. This standard measurement served as a reference point for assessing the fluorescence response of SQ-122 PC to chymotrypsin under the specified conditions, providing a quantitative basis for evaluating enzyme interaction with the probe.

### 3.3. Determination of Detection Limit in Chymotrypsin Assays

The concentration of the probe (SQ-122 PC) was 5 μM in H_2_O (30% DMSO). Chymotrypsin concentrations ranged from 0.025 nM to 0.5 nM. The fluorescence recovery of each substrate was assessed after 60 min by incubating at 37 °C at emission maxima of 669 nm. The detection limit for chymotrypsin was determined using the subsequent formula [46]:

LOD = 3.3 (SD)/S

where SD represents the standard deviation of the sample, and S denotes the slope derived from the linear part of the calibration curve fitting.

### 3.4. Fluorescence Quenching Efficiency

Fluorescence quenching efficiency was calculated using the formula below [47]:

(1) $$\mathrm{Fluorescence}\;\mathrm{quenching}\;\mathrm{efficiency}\;\left(\%\right)=[1-(F_{PC}/F_{dye})]\times 100$$

where *F_PC_* is the emission intensity of SQ-122 PC, and *F_dye_* is the emission intensity of the SQ-122 dye at the same concentration in H_2_O (30%).

## 4. Conclusions

In summary, we designed and successfully synthesized a novel squaraine dye-based far-red fluorescent probe that can selectively detect chymotrypsin activity with very high sensitivity in the far-red wavelength region. The turn-on of FRET in the probe led to a quenching of fluorescence intensity. However, the incubation of the probe with the enzyme led to a tremendous enhancement of fluorescence emission in the far-red region due to FRET-off. The probe showed high sensitivity with a very low detection limit of 0.130 nM and good selectivity towards chymotrypsin in the presence of other competitive enzymes. The implications of this research extend beyond the immediate findings, suggesting a versatile framework for developing far-red probes tailored to the detection of specific enzymatic activities, thereby enhancing the capabilities of biochemical analysis and diagnostic procedures.

## Figures and Tables

**Table 1:** Photophysical parameters of SQ-122 and SQ-122 PC in DMSO and H_2_O (30% DMSO).

DMSO					H_2_O (30% DMSO)			
λ_abs_		λ_em_	Δ	ε (dm^3^ mol^−1^ cm^−1^)	λ_abs_	λ_em_	Δ	ε (dm^3^ mol^−1^ cm^−1^)
SQ-122	667 nm	682 nm	15 nm	1.85 × 10^5^	659 nm	669 nm	10	0.62 × 10^5^
SQ-122 PC	667 nm	682 nm	15 nm	1.95 × 10^5^	662 nm	669 nm	7	0.68 × 10^5^

**Table 2:** Comparison of SQ-122 PC with previously reported work for CHT detection.

Methods	Probe	λ_ex_/λ_em_ (nm)	Far-Red	LOD	Reference
Turn-on fluorescence	Probe	450/515	No	50 ng/mL	[42]
Ratiometric Fluorescent	NI	385/450 and 550	No	0.34 nM	[43]
Turn-on fluorescence	CyB	670/695	Yes	0.5 nM	[44]
Turn-on fluorescence	PPESO_3_	440/520	No	6 pM	[45]
Turn-on fluorescence	SQ-122 PC	662/669	Yes	0.13 nM	This work

## Data Availability

The data presented in this study are available on request from the corresponding author. The data are not publicly available due to ethical and privacy reasons.

## Supplementary Materials

The following supporting information can be downloaded at: https://www.mdpi.com/article/10.3390/molecules29061282/s1, Scheme S1: Synthetic details, along with TOF-MS, HPLC, ^1^HNMR, and ^13^CNMR characterizations; Figure S1: Electronic absorption spectra of SQ-122 and SQ-122 PC in 5 μM (a) DMSO solution and in (b) H_2_O (30% DMSO); Figure S2: Time dependent fluorescence spectra of SQ-122 PC towards chymotrypsin (0.5 nM); Figure S3: TOF-MS of SQ-122 PC after enzymatic cleavage of the probe.
