# Supplementary material for: Design and Synthesis of Novel Squaraine-Based Fluorescent Probe for Far-Red Detection of Chymotrypsin Enzyme
Source: Molecules. 2024 Mar 14;29(6):1282. doi: 10.3390/molecules29061282 (PMC10975582; doi:10.3390/molecules29061282)
Supplement: Supplementary file 1 [file molecules-29-01282-s001.zip › molecules-2888805-supplementary.pdf]

Design and Synthesis of Novel Squaraine-Based Fluorescent Probe for NIR Detection of Chymotrypsin Enzyme

Shekhar Gupta, Priyanka, Saikiran Mavileti, Shyam S. Pandey and Tamaki Kato

Graduate School of Life Science and Systems Engineering, Kyushu Institute of Technology, 2-4 Hibikino, Wakamatsu, Kitakyushu 808-0196, Japan

Email: guptashekhar176@gmail.com, shyam@life.kyutech.ac.jp, tmkato@life.kyutech.ac.jp

Experimental

S1. Synthesis of Unsymmetrical Squaraine dye SQ122

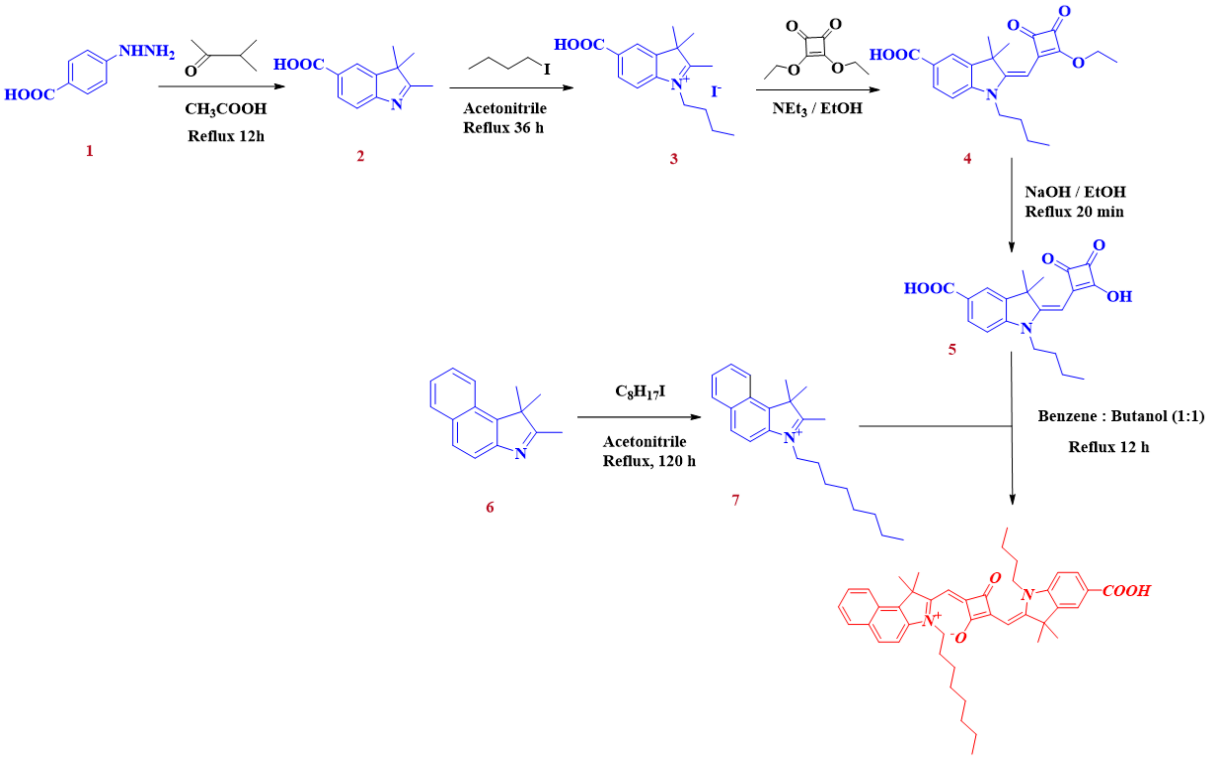

Scheme S1. *Scheme for synthesis for unsymmetrical squaraine dye SQ-122.*

S1.1. Synthesis of 2,3,3-trimethyl-3H-indole-5-carboxylic acid (2)

2,3,3-trimethyl-3H-indole-5-carboxylic acid was synthesized following the procedure reported by Pandey et al. [1] **TOF-mass**: $C_{12}H_{13}NO_2$ (calculated *m/z* = 203.0946 and measured m/z = 204.1028 $[M+H]^+$).

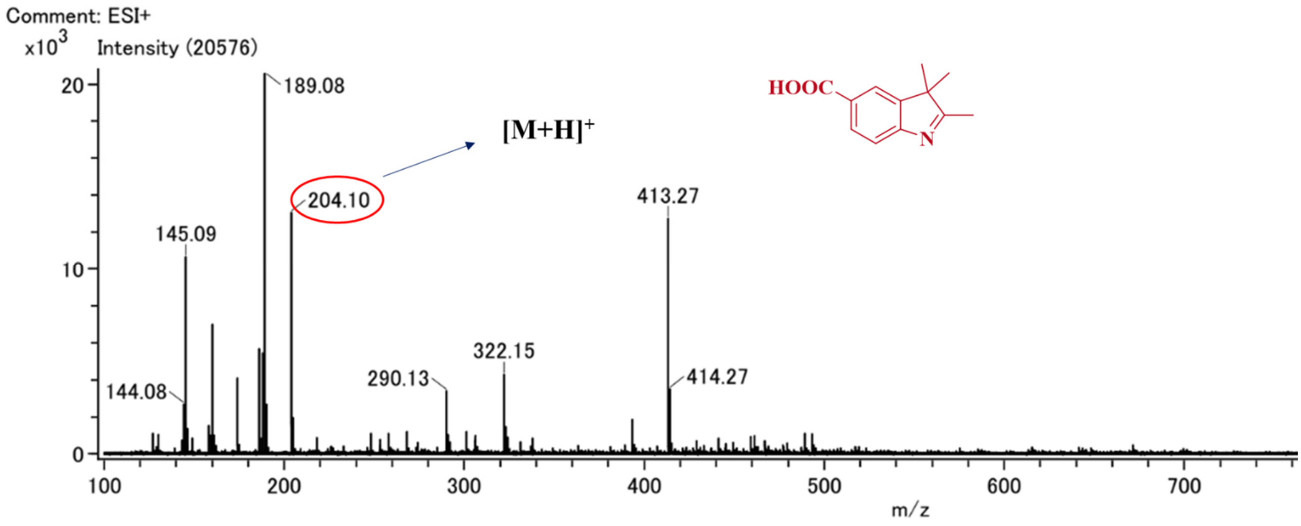

TOF-MASS of **Compound 2**

S1.2. Synthesis of 1-butyl-5-carboxy-2,3,3-trimethyl-3H-indolium (3)

2,3,3-trimethyl-3H-indole-5-carboxylic acid (1 equiv.) and 1-iodobutane (3 equiv.) were dissolved in acetonitrile and reaction was refluxed for 36 h at 90^0C. The reaction was monitored by TLC using chloroform:methanol (9:1) system. Upon the completion of the reaction, the solvent was evaporated under reduced pressure and the product was precipitated with ample addition of ether. The solid was filtered and dried, yielded 81%. **TOF-mass** (measured 260.17 $[M]^+$; calculated 260.16451).

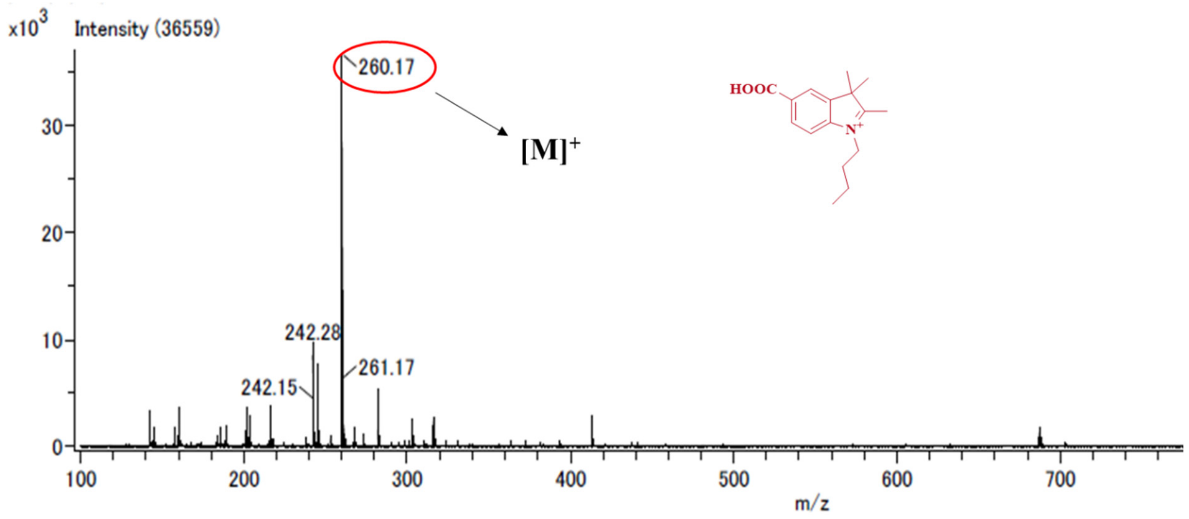

TOF-Mass of **Compound 3**

S1.3. Synthesis of (E)-1-butyl-2-((2-ethoxy-3,4-dioxocyclobut-1-en-1-yl)methylene)-3,3-dimethylindoline-5-carboxylic acid (semi squaraine ester) (4)

In a round bottom flask with a condenser, 1-butyl-5-carboxy-2,3,3-trimethyl-3H-indolium (5 g; 19 mmol), diethoxy squarate (3.4 g; 20 mmol), and trimethylamine (2 ml) were added and dissolved in ethanol. The reaction mixture was then refluxed for 1 hour. The progress of the reaction was monitored by TLC. Upon the completion of the reaction, as monitored by TLC, the solvent was removed under reduced pressure. The crude was then purified by silica gel column chromatography yielded 60%. **TOF-Mass** (measured 384.81 $[M+H]^+$ and 406.16 $[M+Na]^+$; calculated 383.18327 $[M+H]^+$).

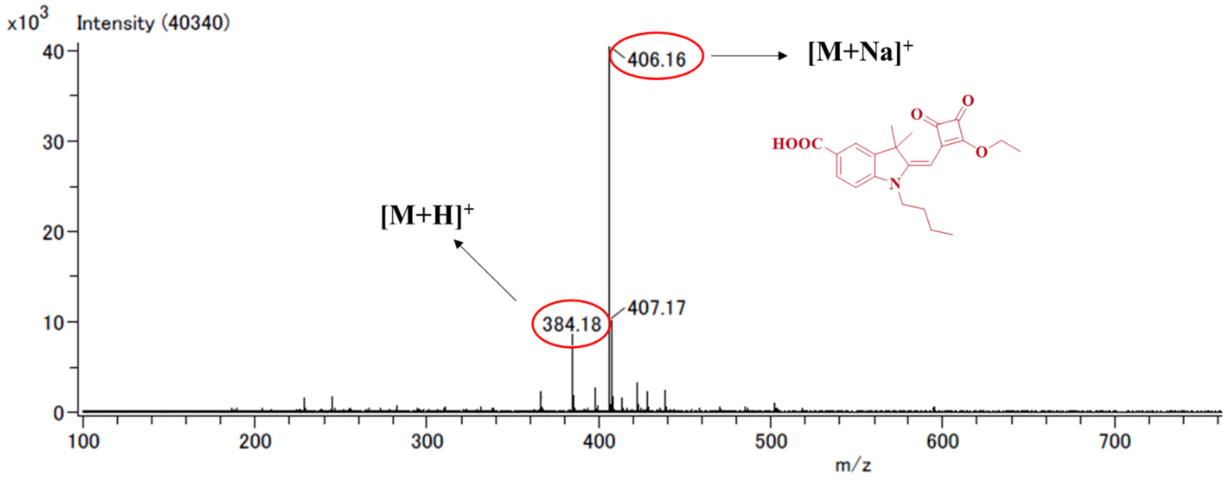

TOF-Mass of **Compound 4**

S1.4. Synthesis of 1,1,2-trimethyl-3-octyl-1H-benzo[e]indol-3-ium (7)

5g (1 equiv.) of 1,1,2-trimethyl-1H-benzo[e]indole **(6)** and 17.2 g (3 equiv.) of 1-iodoctane were dissolved in 50 mL of acetonitrile taken in a round-bottomed flask fitted with a condenser. The reaction mixture was refluxed for 120 hours. Reaction monitoring was done by TLC using Hexane: Ethyl acetate (1:1) as the eluting solvent. After completion of the reaction as confirmed by TLC, the solvent was evaporated under vacuum. The product was precipitated with hexane and the solid obtained was filtered under vacuum and dried under vacuum. **TOF-Mass** (322.84 measured $[M+H]^+$; calculated 322.25293 $[M+H]^+$)

S1.5. Synthesis of unsymmetrical squaraine dye (SQ122)

Semi squaraine ester (1 gm; 2.6 mmol) was taken in a round bottom flask and dissolved in 30 ml of ethanol. To this reaction mixture 40 % NaOH (0.6ml) was added and refluxed at 100^0C for 30 minutes. The reaction progress was monitored by TLC. Upon the completion, the reaction mixture

was cooled and 1.2 ml of 20 % HCl was added. Upon cooling, the solvent was evaporated under reduced pressure, to this residue 1,1,2-trimethyl-3-octyl-1H-benzo[e]indol-3-ium (676.72 mg; 2.1 mmol) and 1:1 ratio of benzene/butanol was added. The reaction mixture was then refluxed at 110^0C for 12 hours. The reaction was monitored by TLC and HPLC. After the reaction, the solvent was evaporated and the crude was purified by employing silica gel column chromatography yielded 60%. **TOF-Mass** (measured 659.39 $[M+H]^+$; 658.37706 calculated). **1H NMR** (500 MHz, $CDCl_3$): δ /ppm = 8.22 (d, 1H, CH_{arom}); 8.12 (d, 1H,CH_{arom}); 8.0 (s,1H, CH_{arom}); 7.9(t,1H, CH_{arom}); 7.61 (t,1H,CH_{arom}); 7.48 (t,1H,CH_{arom}); 7.35 (d,1H,CH_{arom}); 6.16 (s,1H,C$H_{methine}$); 6.03 (s,1H, C$H_{methine}$); 4.21 (t,2H,N-C$H_{methylene}$); 3.97 (t,2H,N-C$H_{methylene}$); 2.96 (s,6H,CH_{methyl}); 2.89 (s,6H, CH_{methyl}) ; 1.90-1.77 (m, 4H,$CH_{methylene}$) ; 1.51-1.25 (m,12H, $CH_{methylene}$); 1.0 (t, 3H, CH_{methyl}); 0.86 (t, 3H, CH_{methyl}) and **13C NMR** (500 MHz, $CDCl_3$) : δ /ppm = 181.52, 176.20, 173.99, 170.22, 167.85, 147.19, 141.99, 139.15, 135.39, 131.16, 129.88, 129.74, 128.53, 127.54, 124.87, 124.01, 123.80, 122.72, 110.35, 108.21, 87.80, 87.35, 51.83, 48.32, 44.28, 43.43, 31.71, 29.31, 29.13, 29.01, 27.60, 27.31, 27.01, 26.58, 22.59, 20.38, 14.06 and 13.89 confirms the identity of the synthesized product.

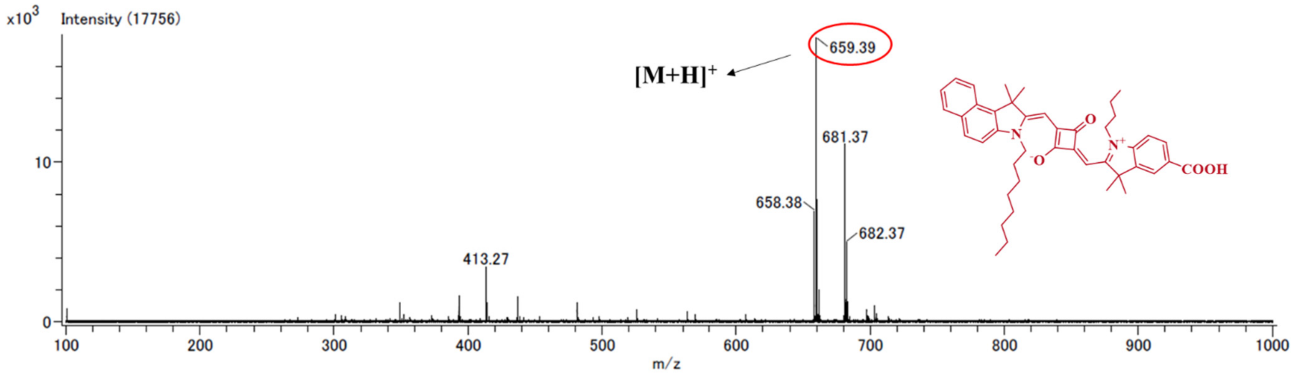

TOF-Mass of ***SQ-122***

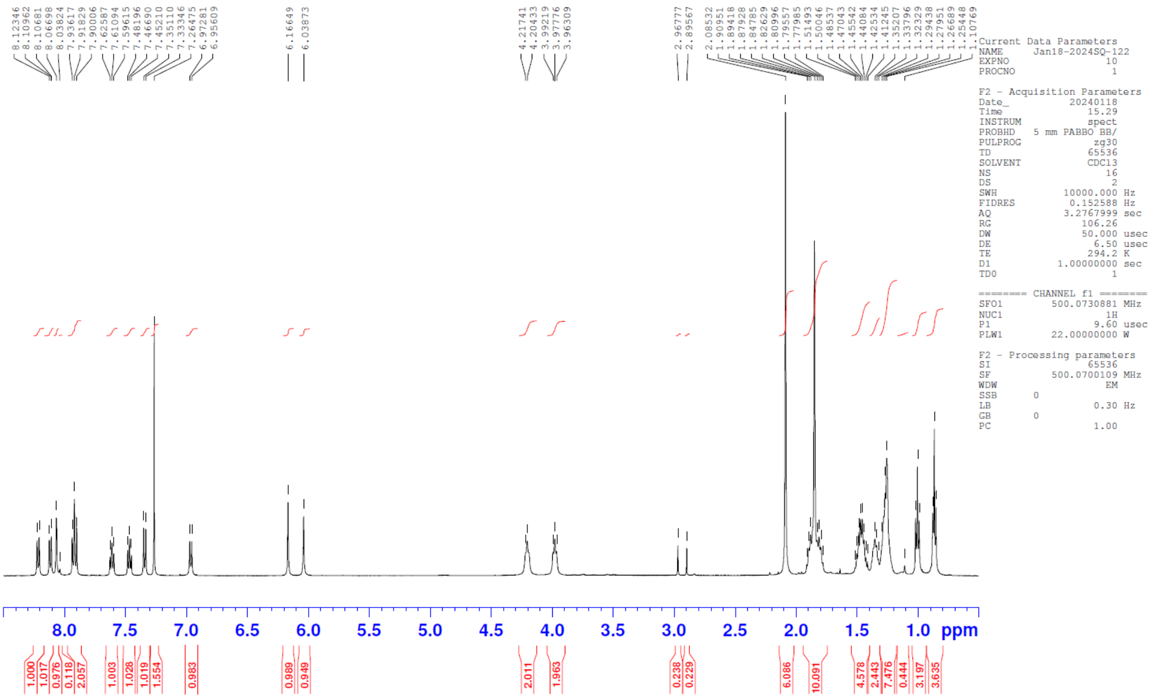

^{1}H NMR of ***SQ-122***

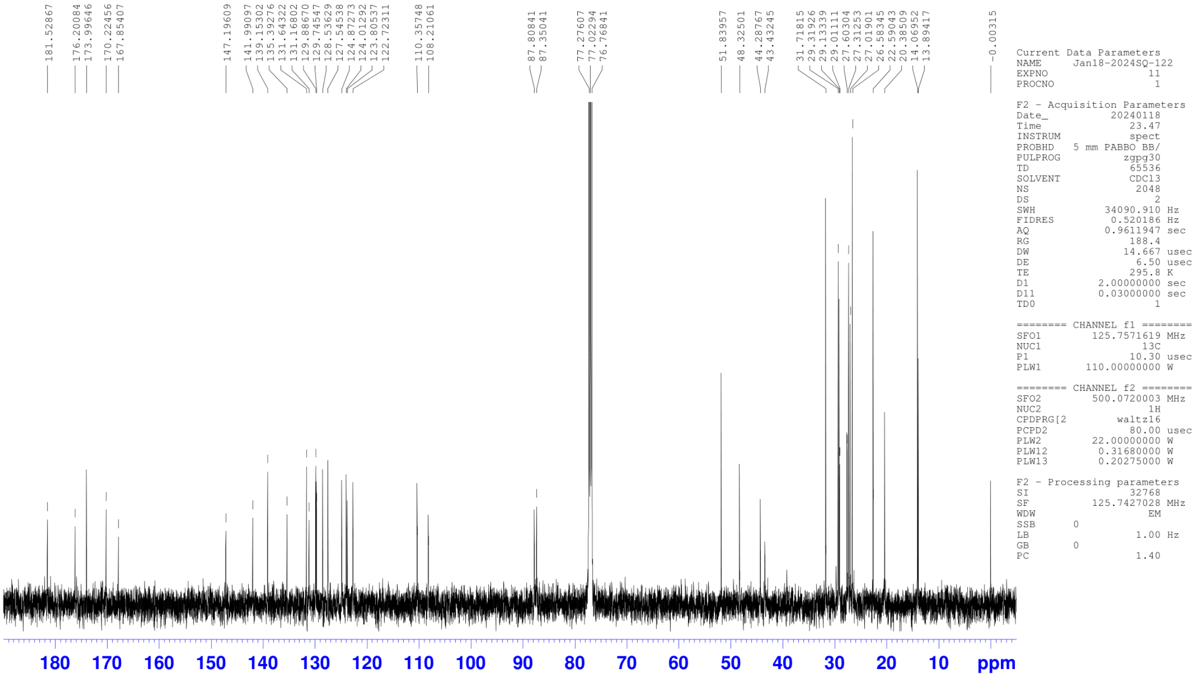

^{13}C NMR of ***SQ-122***

S1.6. Solid Phase Synthesis of Peptide Sequence

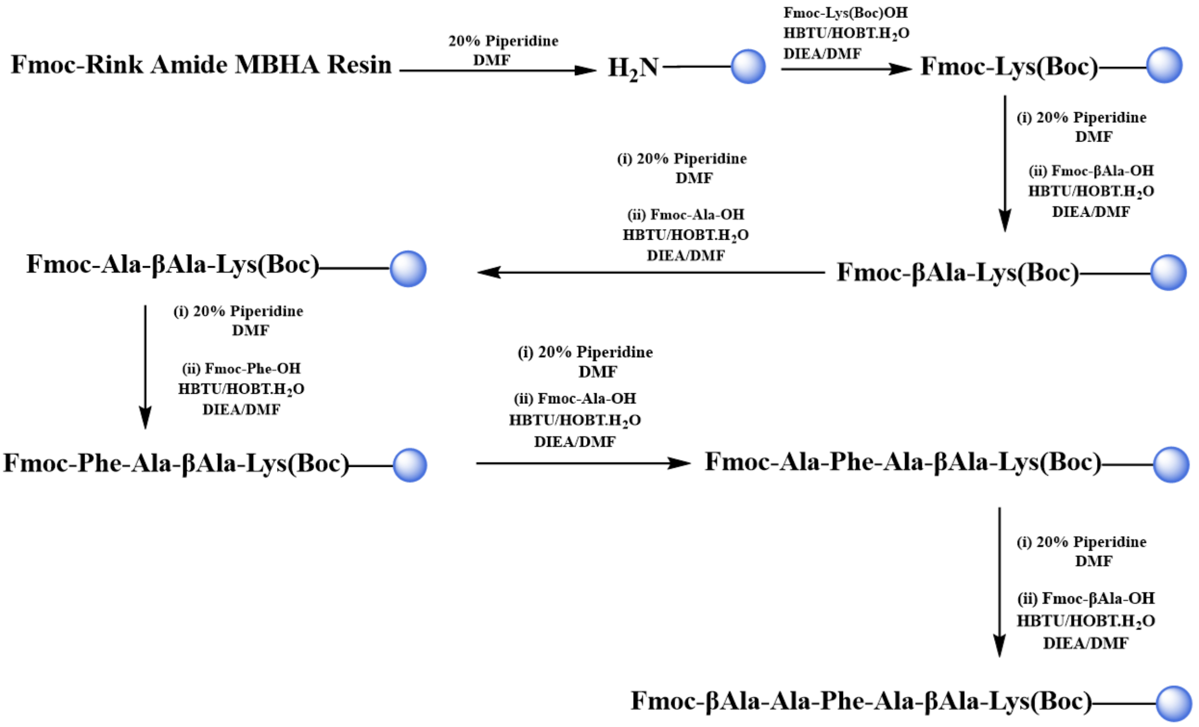

Scheme S2. *Scheme for synthesis of peptide sequence Fmoc-βAla-Ala-Phe-βAla-Lys (Boc)-Resin.*

Fmoc-Lys (Boc)OH was loaded onto Rink Amide MBHA resin (substitution 0.41 mmol/g resin) using Fmoc/piperidine strategies on a 0.432 mmol scale. HBTU and HOBT.H_2O were used as activating agents. Subsequently Fmoc-β-Ala-OH, Fmoc-Ala-OH, Fmoc-Phe-OH, Fmoc-Ala-OH and Fmoc-β-Ala-OH were anchored. Small amount of the corresponding resin-supported peptide was subjected to TFA/Triisopropylsilane/H_2O = 95:2.5:2.5 and the resin-supported peptide was

cleaved from the resin in the cleavage cocktail. The peptide was precipitated by ether in an ice bath. The crude peptide was purified by HPLC. The purified substrate was analyzed by HR-MS (ESI-TOF-MS). TOF Mass (measured 799.42 $[M+H]^+$; 798.40646 calculated).

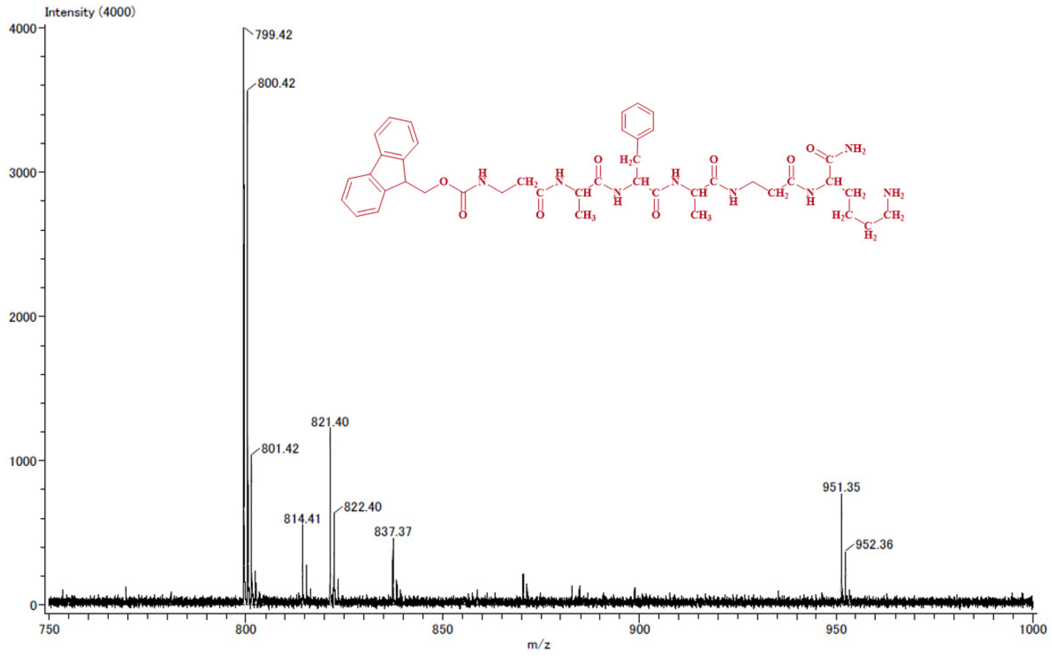

TOF-Mass of *Fmoc-βAla-Ala-Phe-βAla-Lys-NH₂*

S1.7. Synthesis of Dye Peptide Conjugate (SQ-122 PC)

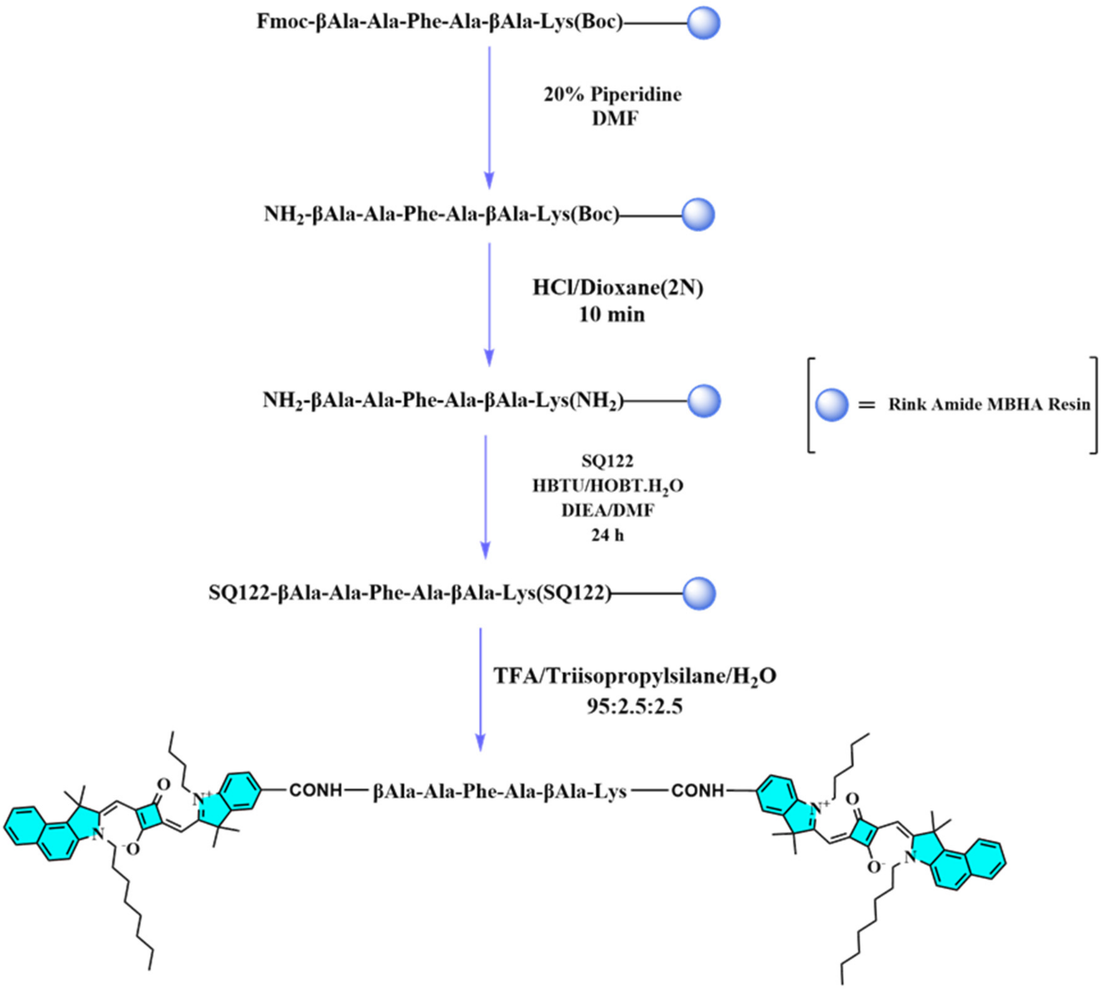

Scheme S3. *Scheme for synthesis of dye peptide conjugate SQ122-βAla-Ala-Phe-βAla-Lys-SQ122*

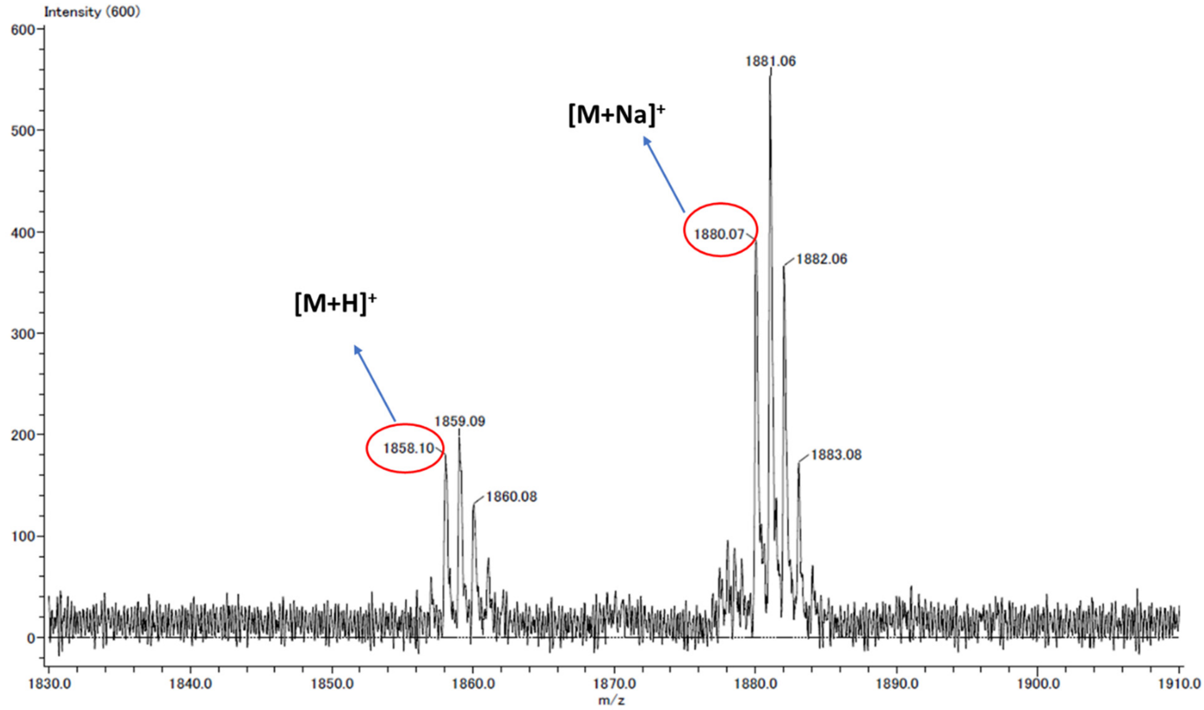

TOF-Mass of *SQ-122 PC*

CH. 2 C.S 2.50 ATT 8 OFFS 0 00/00/00 01:22

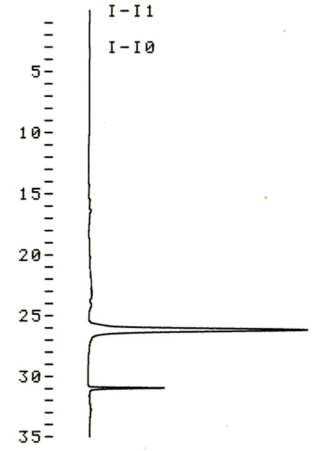

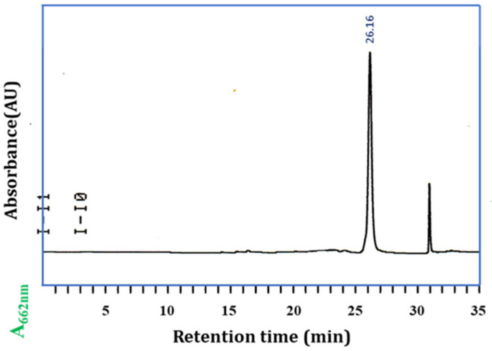

D-2500 00/00/00 01:22

METHOD: TAG: 15 CH: 2

FILE: 1 CALC-METHOD: AREA% TABLE: 0 CONC: AREA

NO.	RT	AREA	CONC	BC
10	26.16	1463952	90.100	BB
11	30.94	172142	9.900	BV
TOTAL				
		1738823	100.000	

PEAK REJ : 100000

HPLC of *the probe SQ-122 PC.*

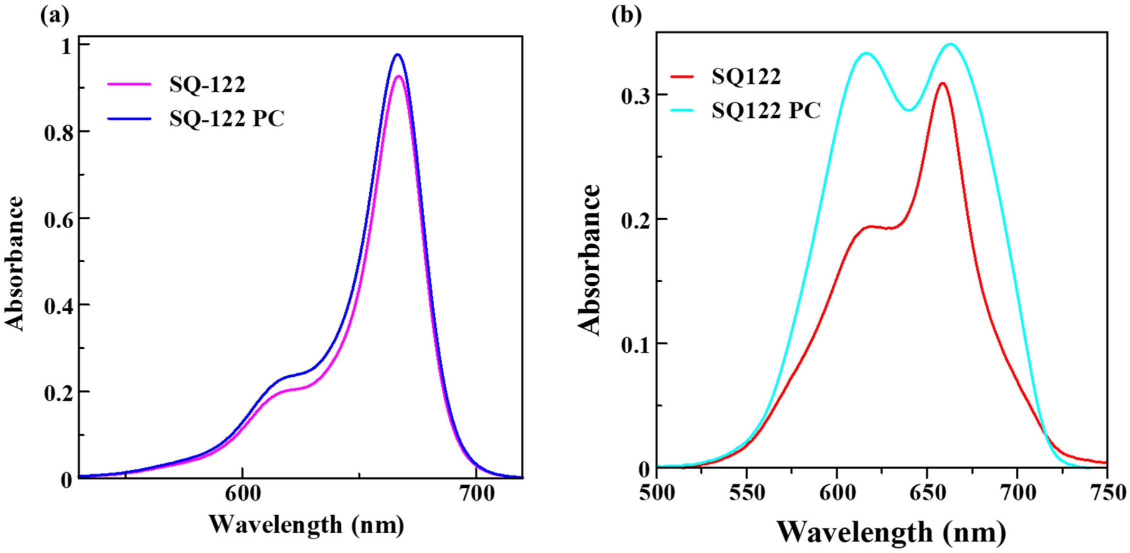

Figure S1. UV–visible–near-infrared (NIR) electronic absorption spectra of SQ-122 and SQ-122 PC in 5μM **(a)** DMSO solution and in **(b)** H_2O (30% DMSO).

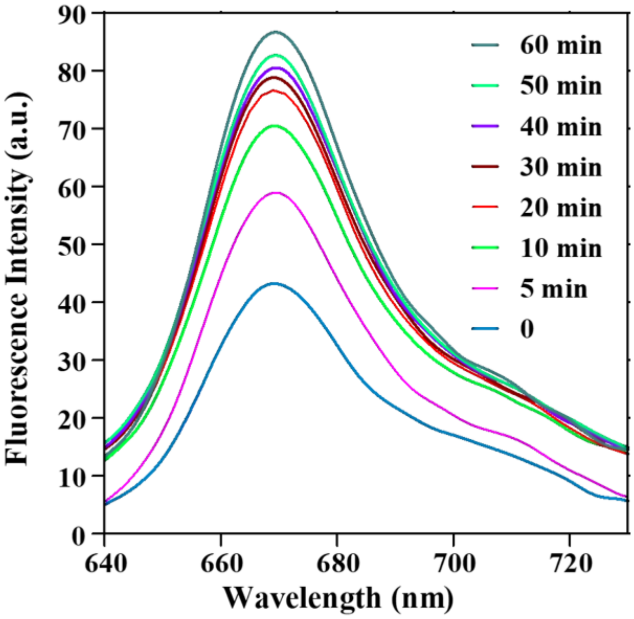

Figure S2. Time dependent fluorescence spectra of SQ-122 PC, Probe towards chymotrypsin. The concentration of chymotrypsin is 0.5nM; reaction occurred at 37°C for 60 min in H_2O (30% DMSO as co-solvent).

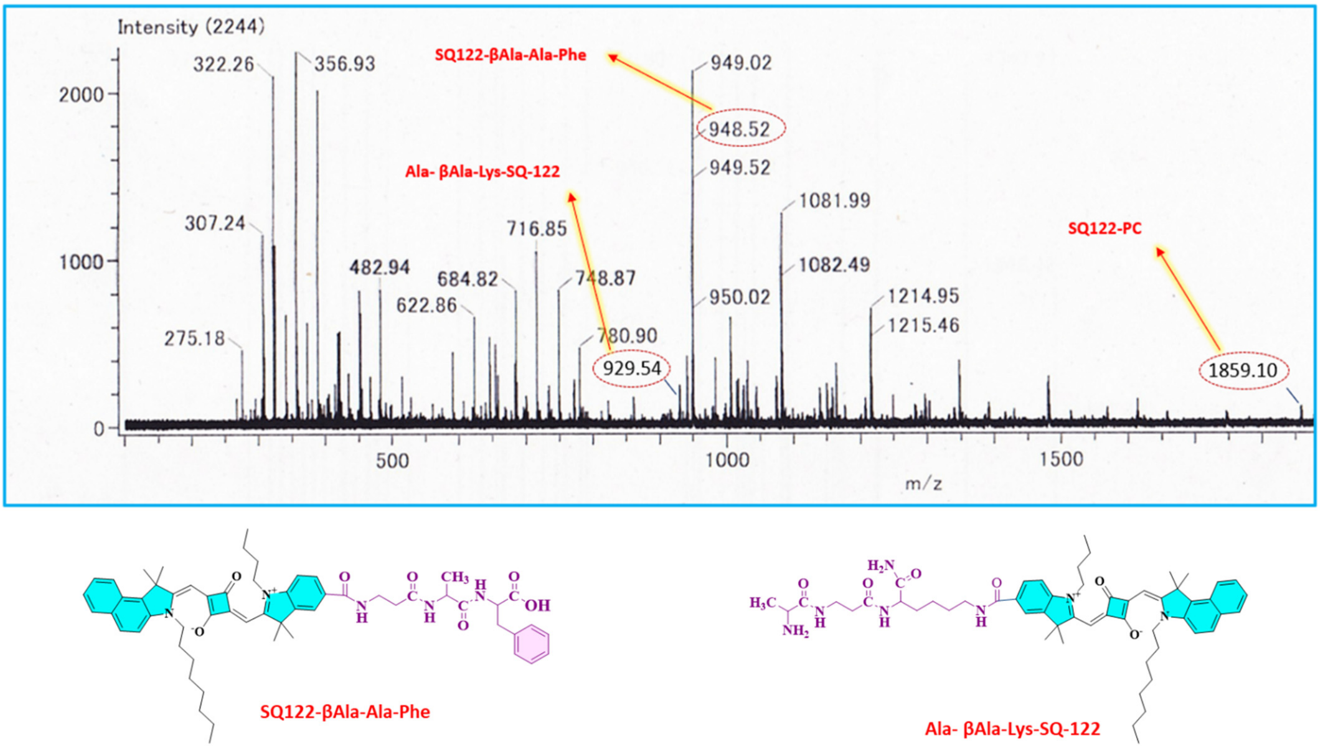

Figure S3. TOF-MS of SQ-122 PC after enzymatic cleavage of the probe. The concentration of enzyme was 10nM in 10μM of probe in H_2O (30% DMSO) incubated at 37°C for 60 min.

References:

1. Saikiran, M.; Pandey, S.S.; Hayase, S.; Kato, T. Photophysical Characterization and BSA Interaction of Direct Ring Carboxy Functionalized Symmetrical Squaraine Dyes. *J. Phys. Conf. Ser.* **2017**, *924*, 12006, doi:10.1088/1742-6596/924/1/012006.
